# Exploring the efficacy of GRU model in classifying the signal to noise ratio of microgrid model

**DOI:** 10.1038/s41598-024-66387-1

**Published:** 2024-07-06

**Authors:** Abdulaziz A. Alsulami, Qasem Abu Al-Haija, Badraddin Alturki, Ali Alqahtani, Faisal Binzagr, Bandar Alghamdi, Rayan A. Alsemmeari

**Affiliations:** 1 Department of Information Systems, Faculty of Computing and Information Technology, King Abdulaziz University, 21589 Jeddah, Saudi Arabia; 2 Department of Cybersecurity, Faculty of Computer & Information Technology, Jordan University of Science and Technology, PO Box 3030, Irbid, 22110 Jordan; 3 Department of Information Technology, Faculty of Computing and Information Technology, King Abdulaziz University, 21589 Jeddah, Saudi Arabia; 4 Department of Networks and Communications Engineering, College of Computer Science and Information Systems, Najran University, 61441 Najran, Saudi Arabia; 5 Department of Computer Science, Faculty of Computing and Information Technology, King Abdulaziz University, P.O. Box 344, 21911 Rabigh, Saudi Arabia

**Keywords:** Energy science and technology, Engineering

## Abstract

Microgrids are small-scale energy system that supplies power to homes, businesses, and industries. Microgrids can be considered as a trending technology in energy fields due to their power to supply reliable and sustainable energy. Microgrids have a mode called the island, in this mode, microgrids are disconnected from the major grid and keep providing energy in the situation of an energy outage. Therefore, they help the main grid during peak energy demand times. The microgrids can be connected to the network, which is called networked microgrids. It is possible to have flexible energy resources by using their enhanced energy management systems. However, connection microgrid systems to the communication network introduces various challenges, including increased in systems complicity and noise interference. Integrating network communication into a microgrid system causes the system to be susceptible to noise, potentially disrupting the critical control signals that ensure smooth operation. Therefore, there is a need for predicting noise caused by communication network to ensure the operation stability of microgrids. In addition, there is a need for a simulation model that includes communication network and can generate noise to simulate real scenarios. This paper proposes a classifying model named Noise Classification Simulation Model (NCSM) that exploits the potential of deep learning to predict noise levels by classifying the values of signal-to-noise ratio (SNR) in real-time network traffic of microgrid system. This is accomplished by initially applying Gaussian white noise into the data that is generated by microgrid model. Then, the data has noise and data without noise is transmitted through serial communication to simulate real world scenario. At the end, a Gated Recurrent Unit (GRU) model is implemented to predict SNR values for the network traffic data. Our findings show that the proposed model produced promising results in predicting noise. In addition, the classification performance of the proposed model is compared with well-known machine learning models and according to the experimental results, our proposed model has noticeable performance, which achieved 99.96% classification accuracy.

## Introduction

**Figure 1:**
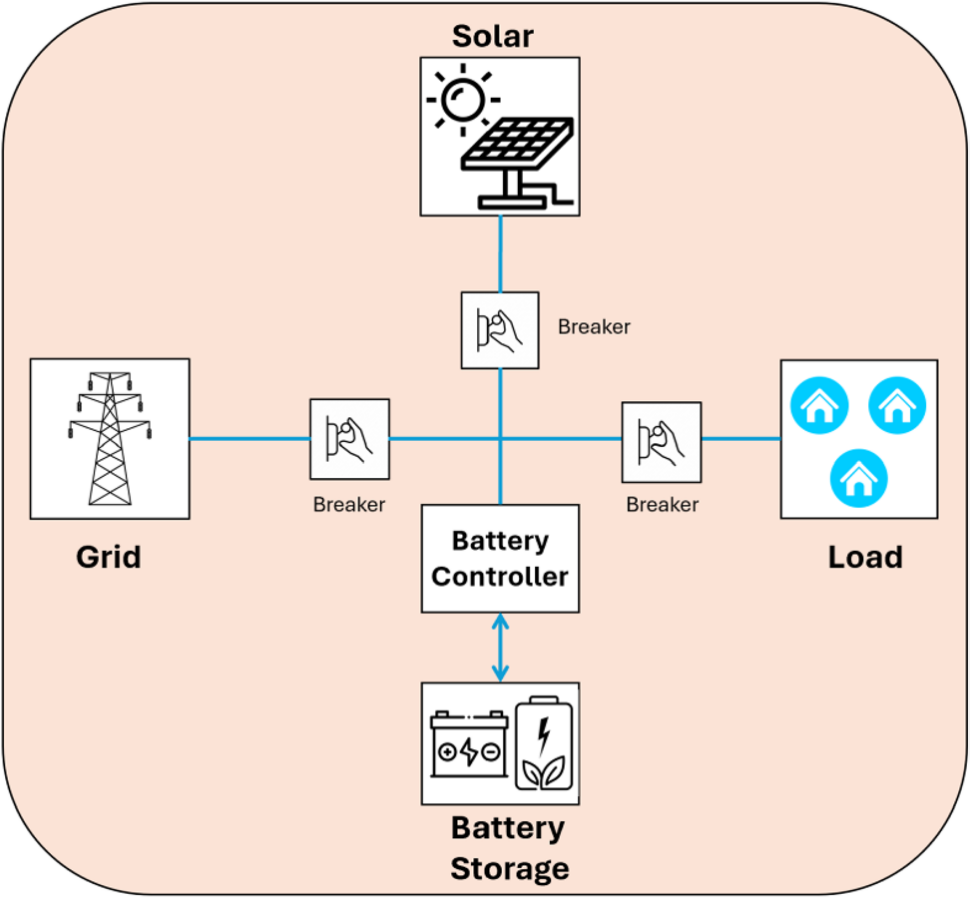
Microgrid model.

Microgrid is one of the trending topics in energy due to the increased demand for energy systems that have reliability and sustainability^1^. Microgrid models can efficiently generate energy in small-scale and localized power systems while maintaining the storage and control of the loads^2^. They work isolated from the main electric grid and provides sustainable, inexpensive power to users, industries, and buildings^3^. To reduce the dependability on centralized power providers and security regarding energy, microgrids became one of the most demanding energy systems. Microgrids can include various sources of renewable power to provide a balance of renewable energy. In addition, they have a main controller system that can manage the creation of multiple energy sources and maintain the energy requirements linked to the loads. Therefore, the cost and usage of energy are reduced^4^. Figure 1 shows a general architecture of a microgrid model, consisting of the following major components solar panels, grid, load, battery storage, and battery controller. Solar panels are used to generate electricity from sunlight. The grid is the main power supply and is used as needed. Load refers to the electricity consumers, which can be houses, businesses, and so on. Battery storage stores the electricity generated by the solar panels. The battery controller manages the distribution of the electricity by controlling the power flow between the solar panels, grid, battery storage, and the load. Adding microgrids to the energy sector and area can be more beneficial than traditional grids. The benefits of microgrids are summarized by authors in^5^, and the discussion of the benefits includes the authors’ thoughts, and some of the additional points are discussed as follows. First, microgrids are flexible and reliable as they can operate isolated from the main energy source, which is called island mode, as they generate and store their energy, which is important in cases of emergency when the power is down. Second, microgrids can be considered green energy and sustainable options when needed as microgrids combine various renewable energy sources containing wind turbines and solar panels. Additionally, windmills, photovoltaic (PV) cells, diesel generator sets, small hydropower, and energy-storing systems can be part of microgrids^6,7^. Third, microgrids have a central management system that balances the energy requirements and loads and maintains the production from various sources, effectively using energy sources and reducing energy costs. Fourth, the microgrids can complement the main electric grid by providing additional capacity when there is high usage and demand and help decrease the loads on the main central grid. Furthermore, microgrids are less independent on the central energy systems. They are usually owned by local facilities and communities, meaning more control over the energy supply can be obtained. Integrating renewable energy sources with microgrids or utility grids is accessible due to enhancements of semiconductor power devices. There are three main kinds of microgrids: direct current (DC) microgrid, alternating current (AC) microgrid, and hybrid microgrids^8^. The DC microgrid is to provide essential loads such as billing counters and computers. At the same time, AC microgrid can supply all loads in both on-grid and off-grid situations. The hybrid microgrid has the DC and AC characteristics^9^.

Recently, AC, DC, and hybrid microgrids became trending platforms for improving the architecture of energy systems. Selecting the architecture depends on the community or facility’s needs and requirements. Several variants of microgrid emerged in recent years for energy storage systems (ESS). Clustering microgrids^10^ is one of the examples that works to group the microgrids depending on shared resources in proximity. The main goal of clustering microgrids is to create performance optimization and collaboration among the microgrids that are in the group. This architecture can help improve load balancing, system efficiency, and resource sharing. Another variant of microgrid is multi-energy microgrid^11^, which accommodates many energy demands. Networked microgrid^12^ which is interconnected and exchanges information and power. The interconnection of microgrids will allow them to cooperate, which will help enhance the resilience and efficiency of the network. These types of girds can be helpful in the optimization of resource allocation and system performance improvements. Another type of microgrid, aviation or shipboard, is designed to serve aviation and maritime applications. These microgrids concentrate on efficiently supplementing power, usage of energy, and system resilience in challenging environments like airplanes and ships.

There are two modes that the microgrids can operate, including standalone and grid connected. In the grid connected, microgrids remain connected to the main grid for receiving and sending the energy as required. When there is an error or malfunction in the main grid, the microgrids shift to the other standalone mode and keep powering important loads. This can be accomplished by detaching the whole microgrid from the main grid or some feeders inside the microgrids. When all grids are disconnected, all the micro sources should provide power to all the loads in the feeders. When some feeders are detached, they provide energy to loads while others escape the interruption^13^.

The power management tasks in microgrids ensure that the microgrids are efficient in several situations and scenarios based on various aspects, including load and weather prediction, the state of the device, and the use of distributed energy resources. Several power management systems proposed in academia with economic, environmental, and technical drawbacks^14–19^.

By using the networked microgrids, there is a possibility of reaching flexibility among power resources by including the enhanced power management system of microgrids^20,21^. Microgrids can produce extra energy to exchange with different microgrids that can be out of energy, or maybe microgrids want to purchase power from the major grid, this will help reduce transmission losses and grid outages prevention; these can be achieved by the help of various power conversion techniques^22,23^. In networked microgrid systems, two operational states can be seen: normal operation and emergency operation. The networked microgrid system connects to the main energy distribution network during normal operation. During emergency, the networked microgrid system operates after an outage of energy or working independently; in this state, the service is restored by starting the grid.

Several challenges can arise with communication over the network, including controlling and monitoring the networked microgrid systems, as there will be different controllers with various degrees of independence, as well as the noise that will appear due to the communication over the network. There are several types of noise in the industrial environment, including measurement noise, such as sensors; communication noise, such as antennas; and environmental noise, such as rain^24^. In distributed microgrid control systems, the noise of communication can be considered additive noise taken from antennas in the communication that is considered wireless. Also, the communication noise can be stated to be created at the receiver’s front end. In addition, it can be categorized as thermal noise produced by the electrical devices next to the receivers. Gaussian distribution model based on white noise is a common approach in modelling communication noise^25^. The small variance of Gaussian communication noise can affect the load of electronics, and in the long term, it will minimize the devices’ longevity^25^. However, according to authors in Ref.^26^, the large variance of Gaussian noise will produce current circulation in the microgrid and risk its stability. Communication noise may generate noise on the control signal, such as frequency and voltage droop reference values, which can hurt the control system’s performance. The frequency droop reference signal that has noise will oscillate the inverter bridge’s operating switching frequency. The parameter tuning of proportional-integral controllers in the internal voltage and current control loops of inverters can be impacted by the voltage reference signal that has a noise^27–31^. Furthermore, having a low SNR can be challenging for most researchers, so they did not give it full attention^32^. As a result, a noise management method is required to minimize the noise effect on the control system of the microgrid.

This research proposes a Noise Classification Simulation Model (NCSM) that includes a communication network and can generate noise to simulate real scenarios. In addition, it uses the power of deep learning to detect, classify, and predict noise in real-time communication in microgrid systems. The main contributions of this paper are as follows:

- We apply Gaussian white noise to the collected data to create an emergent dataset and label its classes.
- We implement a serial communication model to transmit sensor data of the microgrid in real-time.
- We explore the power of the GRU model in detecting and classifying SNR values.
- We compare and analyze the performance of GRU with various machine learning models, i.e., long short-term memory (LSTM), conventional recurrent neural network (RNN), decision tree (DT), bagged tree (BT), and shallow neural networks (SNN).

The remainder of this paper is organized as follows: “Literature review” reviews the related works in microgrids. Section “Problem statement” discusses and explains the methodology, dataset, and GRU model. Section “Methodology” provides the experiments, the results, and the evaluation. Section “Experiment and result” concludes our work and outlines future work.

## Literature review

This section first reviews research that focuses on addressing noise in networked microgrids. Second, it demonstrates the benefits of using serial communication in microgrids. Thirdly, it discusses different models used by researchers to detect and classify noise, including statistical methods and machine learning models. Table 1 shows a summary of related works in noise detection.

**Table 1:** A summary of related works in noise detection.

Year	Focus area	Authors (references)	Approach	Results/outcomes
2019	Wide Area Measurement Systems	Chen et al.^62^	S-G filter for de-noising and Adaptive TLS-ESPRIT algorithm with SVAPAIR for order setting	Better adaptability and anti-noise characteristics obtained
2020	Fault Location in DC Microgrid	Nagam et al.^50^	GPR and supervised and semi-supervised learning	Estimate fault resistance
2020	Microgrids Fault Detection and Classification	Fahim et al.^55^	DWT, RBM, and ANN	Improved fault detection and classification accuracy
2021	Microgrids Fault Detection and Classification	Bhuiyan et al.^56^	DBN and DWT	High accuracy in fault detection and classification
2022	HIF Fault Detection and Classification in Microgrids	Biswal et al.^48^	DWT, DT, and summation of accumulated differences of residual voltage	DT outperformed other classifiers
2022	Microgrid Protection	Imran et al.^51^	KM	Detecting Faults
2023	Microgrids Fault Detection and Classification	Alsaba et al.^54^	A hybrid CNN-GRU	Improved fault detection accuracy
2023	Power Quality Disturbance Classification	Cai et al.^53^	CNN-GRU	Achieved high classification accuracy
2023	Fault Detection in Distribution Networks	Gangwar et al.^49^	K-medoids clustering and KNN	Improved accuracy in fault location estimation
2024	Detection in DC Microgrids	Gao et al.^57^	MRC-SDFE, SHO, and DBN	Improved islanding detection

### Noise in networked microgrid systems

Networked microgrids (NMGs) are considered one of the most critical parts of microgrid implementation due to their ability to operate, monitor, and control distributed energy resources. Communication channels typically experience noise interruptions, specifically in noisy environments, which can prevent NMG components from exchanging information effectively^33^. Such disturbances often originate from switching power supplies, silicon-controlled rectifiers, brush motors, dimmer switches, and industrial equipment, all directly connected to the supply network^34^. Several approaches have been developed to overcome noise challenges in microgrid settings to preserve stability and improve efficiency. Authors in Ref^35^ introduced a modern state-space controller using linear quadratic Gaussian (LQG) for connected and islanded microgrids. They worked on enhancing stability by designing a distinct LQG controller to address disturbances and noise. The input signals were injected into the model with white noise, and the framework employed state space methods for the controller’s development, which integrates optimal gain from LQR and a Kalman filter to eliminate the noise. The authors in Ref^36^. presented a multiagent-based nonlinear generalized minimum variance (NGMV) as a controller technique for noise resilience in islanded AC microgrids. The model focuses on voltage and frequency restoration. Utilizing a nonlinear autoregressive second-order Volterra method, the NGMV approach is adept at noise attenuation considering additive white noise. The experiment used two tests with different variance values for voltage and frequency to validate their experiment in MATLAB. Moreover, authors in Refs.^37,38^ introduced resilient distributed regulator models based on the mathematical methodology for islanded AC microgrids. The work in Ref.^37^ considered stochastic noise and delay in communication. The controller algorithm enables voltage and frequency tuning in power-sharing environments. The purpose of the model is to transform the corrupted data into a stability analysis unit that relies on the basic lemmas to control voltage and frequency. However, authors in Ref.^38^ proposed three protocols using algebraic graph theory to maintain the stability of the islanded microgrid network. The experiment and the evaluation include five scenarios to test the controller with noise and time-delay disturbances. However, authors in Ref.^39^ proposed plug-and-play AC/DC resilient distributed control to maintain power stability. In a distributed fixed-time consensus, they added additive noise to all communication channels in microgrid components AC, DC, and interlinking converters. A 6-bus hybrid inverter-based AC/DC microgrid simulation is conducted using MATLAB/Simulink to evaluate the performance of the proposed method. Authors in Ref.^40^ suggested a combination of time domain reflectometry (TDR), the subtractive correlation method (SCM), and multilayer perceptron neural networks to detect faults and noise in aircraft microgrids.

### Serial communication in microgrid systems

Serial communication is an essential interface for managing and controlling microgrids. The benefit of using serial communication model in this research is to exchange sensor data in real-time through several parts of the microgrid, which can enhance the performance and efficiency of the system^41^. Researchers have reported the use of serial communication models in microgrid systems. They focus on the benefit of serial communication in improving the functionalities of monitoring, control, and protection of microgrids. Authors in Ref.^42^ introduced a microgrid model that built based on the concept of Internet of Energy (IoE). An adaptable IoT-based layered framework and added distributed energy resources (DERs) were used with the things layer. The approach identifies each DER in the microgrid as an individual unit and creates two types of communication protocols. Two protocols were used, which are serial communication and peer-to-peer (P2P) networking via Ethernet. In Ref.^43^, the authors proposed a wireless communication model that uses a message queuing telemetry transport (MQTT). It serves as an application layer using the TCP/IP model. It is well-suited for low-bandwidth machine-to-machine (M2M) applications. This lightweight protocol creates a bidirectional information exchange among DERs within a microgrid and a potential implementation of an energy management system (EMS). The proposed model showed the efficiency of serial communication in transmission data, especially in low-power MQTT nodes. Authors in Ref.^44^ proposed a simulation model for a microgrid system implemented in Simulink. It simulates diverse control and optimization strategies. The authors used mixed integer linear programming (MILP) as an example of microgrid cost optimization to validate the model. RS-485 serial communication technology establishes a connection between the energy management system (EMS) and the generators to regulate the battery charging state^45^. The objective is to minimize operating costs while maximizing economic benefits. Microgrid equipment communication protocols and devices were introduced as a hybrid communication infrastructure^46^. It contains three layers that facilitate communication between serial and ethernet interfaces. In addition, authors in Ref.^47^ built a movable measuring device to quantify the power quality in microgrids by adopting IoT capabilities. The serial communication was utilized as a universal asynchronous receiver / transmitter (UART), and web-based tools were offered to access and monitor the data.

### Classification in microgrids

In recent years, microgrid research has seen a surge in utilizing cutting-edge artificial intelligence methodologies to address classification challenges within microgrid systems, spanning from noise classification, fault detection, power quality disturbance (PQD) identification, and others. Various machine learning techniques such as decision tree (DT), deep learning techniques such as and recurrent neural network (RNN), statistical techniques such as Kalman filtering, these techniques have been used effectively in energy system classification.

The authors in Ref.^48^, proposed a machine learning technique to detect high impedance fault (HIF) in microgrid. They used discrete wavelet transform (DWT), decision tree (DT) classification, and the summation of accumulated differences of residual voltage. However, the experiment includes different classifier methods such as k-nearest neighbors (KNN), support vector machine (SVM), and ensemble classifier to compare the performance. The findings demonstrated that the DT algorithm outperforms other classifiers. In Ref.^49^ authors applied a protection strategy for distribution networks that integrate renewable energy, using weighted k-nearest neighbor regression for fault localization and k-medoid clustering for fault detection and classification. The approach demonstrated resilience against Gaussian white noise using two noise levels, and the IEEE 13 & 34 bus system is modified to create a hybrid energy system that utilizes wind and solar power.

Furthermore, authors in Ref.^50^ proposed a statistical technique for identifying faults in DC microgrids using Gaussian process regression (GPR), which uses supervised and semi-supervised learning modes to estimate fault resistance without data preprocessing. Gaussian white noise is added to the signals, four levels of SNRs are applied, and absolute mean error is used to evaluate the performance. However, the GPR model can improve DC microgrid protection systems due to its independence from the microgrid’s operation. In addition, authors in Ref.^51^, developed a protection framework for microgrids employing KM, with dual criteria formulated for fault detection and classification, specifically considering HIF. The KM is vital for analyzing various estimation problems and facilitating optimal state estimation of electrical parameters using a limited number of noisy sampled values within a short interval.

In addition, the classification tasks in the microgrid environment leverage deep learning approaches such as gated recurrent units (GRUs), which is considered a type of recurrent neural network (RNN). The advantage of using these types of networks is the capability of detecting temporal patterns and dependencies in a dynamic environment such as microgrids^52^. One of the works introduced a parallel fusion approach utilizing convolutional neural network (CNN) and GRU to classify PQDs^53^. The attention mechanism focused on using GRU’s hidden states at various times to capture long-term features, while the convolutional layers extract short-term features. Those features acquired from the model were concurrently merged and utilized in the classification process. Within actual NMG infrastructure, various factors tend to create noise in the control cable. For instance, voltage from nearby power cables, switching of primary equipment, lightning transients, and various other factors can originate noise^34^. This type of noise will affect the stability of the microgrid system. Therefore, it is important to have a framework that can detect the noise accurately. In Ref.^54^, authors proposed a hybrid GRU-CNN-directional architecture to detect the faults in AC microgrid. The model applies random Gaussian white noise when the model’s accuracy is assessed. Authors in Ref.^55^ proposed a technique that is based on a deep learning to classify and detect fault signals in microgrids. In their approach, they have integrated DWT and a probabilistic generative model using restricted Boltzmann machines (RBMs) layers. They trained the model with artificial neural network (ANN). It focuses on generating good accuracy in fault classification across several types of noise levels from voltage and signals. They tested the model after utlizing Gaussian white noise with various SNRs. The researchers in Ref.^56^ proposed an approach that is based on deep belief network (DBN) to diagnose the fault in microgrids when working with gaussian white noise to mimic real-world situations. The strategy reduces the impact of noise while detecting fault by integrating DWT for signal processing. The authors simulated the classification model and evaluated with a lower level of SNR. In addition, authors in Ref.^57^ integrated a DBN trained utilizing the sea-horse optimization (SHO) algorithm with multi-scale refined composite standard deviation fuzzy entropy (MRC-SDFE) for identification of islanding and detection of noise in DC microgrids. However, in their experiment, this approach used a feature extraction method and compared two types of noise, Gaussian and fractional. They compared their method with different classification algorithms, which are back propagation neural network (BPNN), stack automatic encoder (SAE), CNN-LSTM and DBN. Each algorithm was tested in the environment with and without noise interference, and the SHO-DBN outperformed other algorithms. Authors in Ref.^58^ suggested using clustering and scoring algorithms in an ensemble approach to anomaly detection in power distribution systems. It introduced a method for anomaly-aware state evaluation that offers improved accuracy in dynamic distribution systems by including outlier identification and correction. The approach was tested with data from the Bronzeville community microgrid to evaluate the real-time anomaly identification using high artificial noise.

Authors in Ref.^59^ study the effect of doubly-fed induction generator (DFIG) in wind farms on system stability of multi-generator power systems with Low Frequency Oscillations (LFOs). They used the Total Least Squares-Estimation of Signal Parameters via Rotational Invariance Techniques (TLS-ESPRIT) algorithm for eigenvalue identification. They evaluated their work by simulation using the IEEE two-area four-machine power system. They achieved good results in increasing the damping ratio of the dominant mode of inter-area oscillation and increasing in the wind power output capacity that raised the damping ratios of intra-area and inter-area oscillations. Authors in Ref.^60^ suggested distributed energy management framework based on Primal–Dual Method of Multipliers (PDMM) and Reinforcement Learning (RL). Also, they used Directed Acyclic Graph (DAG) for secure communication and energy transactions. They used simulation for evaluating their work and their results were promising as they achieved effective energy management while maintaining the computation time and security is enhanced. Furthermore, researchers in Ref.^61^ proposed a multi-layer security protocol combining blockchain technology and reinforcement learning. They used simulation in their evaluation that has smart grid and microgrid under several scenarios of attacks. Their results show that blackouts are reduced and security enhanced. Additionally, authors in Ref.^62^ proposed novel method S-G filter for de-noising and Adaptive TLS-ESPRIT algorithm with Singular Value Accumulation Percentage Adjacency Increment Ratio (SVAPAIR) for order setting. They tested their work using the IEEE four-generator two-area system and actual LFO data from the North American power grid. Based on their results that LFO noise reduced, precisely determination of LFO order and better adaptability and anti-noise characteristics obtained when compared to methods that are conventional. In addition, researchers in Ref.^63^, suggested Wavelet soft-threshold de-noising for noise attenuation and improved Matrix Pencil (MP) algorithm with Ratio of Adjacent Singular Entropy Increment Difference (RASEID) for adaptive order determination. Their evaluation includes three case studies including synthetic signal analysis, IEEE four-generator two-area system, and actual LFO data from the North American power grid. According to their results that LFO noise is attenuated and the MP algorithm’s accuracy is enhanced.

It is clear from the literature that there have been improvements in the field of detecting and classifying noise in microgrids. Researchers have proposed several methods based on machine learning to predict noise. However, there is still an open challenge in classifying and predicting noise in microgrids in real-time. Therefore, our research addresses this challenge by transmitting microgrid data with noise and without noise using serial communication model. In addition, a GRU model is implemented to predicate noise of the network traffic data.

## Problem statement

In this section, we detail the problem formulation of our work by providing the objectives and challenges that our model will address. The details include a concise description of the problem, challenges, and the needs for this research related to microgrid systems. A networked microgrid is a microgrid that includes a communication network between its components to maintain the distribution of energy and stability. Every microgrid has sources of renewable energy, loads, storage, controllers and can work with the main grid autonomously. Microgrids use sensors to measure the dynamics of the microgrid model, such as solar power, secondary power, power load, battery power, and battery state of charge. Noise interference with communication network in networked microgrids can be considered critical when aiming to transmit sensor readings in reliable way. To explore these issues, there is a need to find a microgrid simulation model that can generate sensor readings in real-time. In addition, generating noise is needed on the sensor data to study its impact on the system. Integrating a communication network is essential to transmit sensor data in real-time. Finally, the use of predictive model in networked microgrids is needed to classify noise to mitigate their impacts.

## Methodology

**Figure 2:**
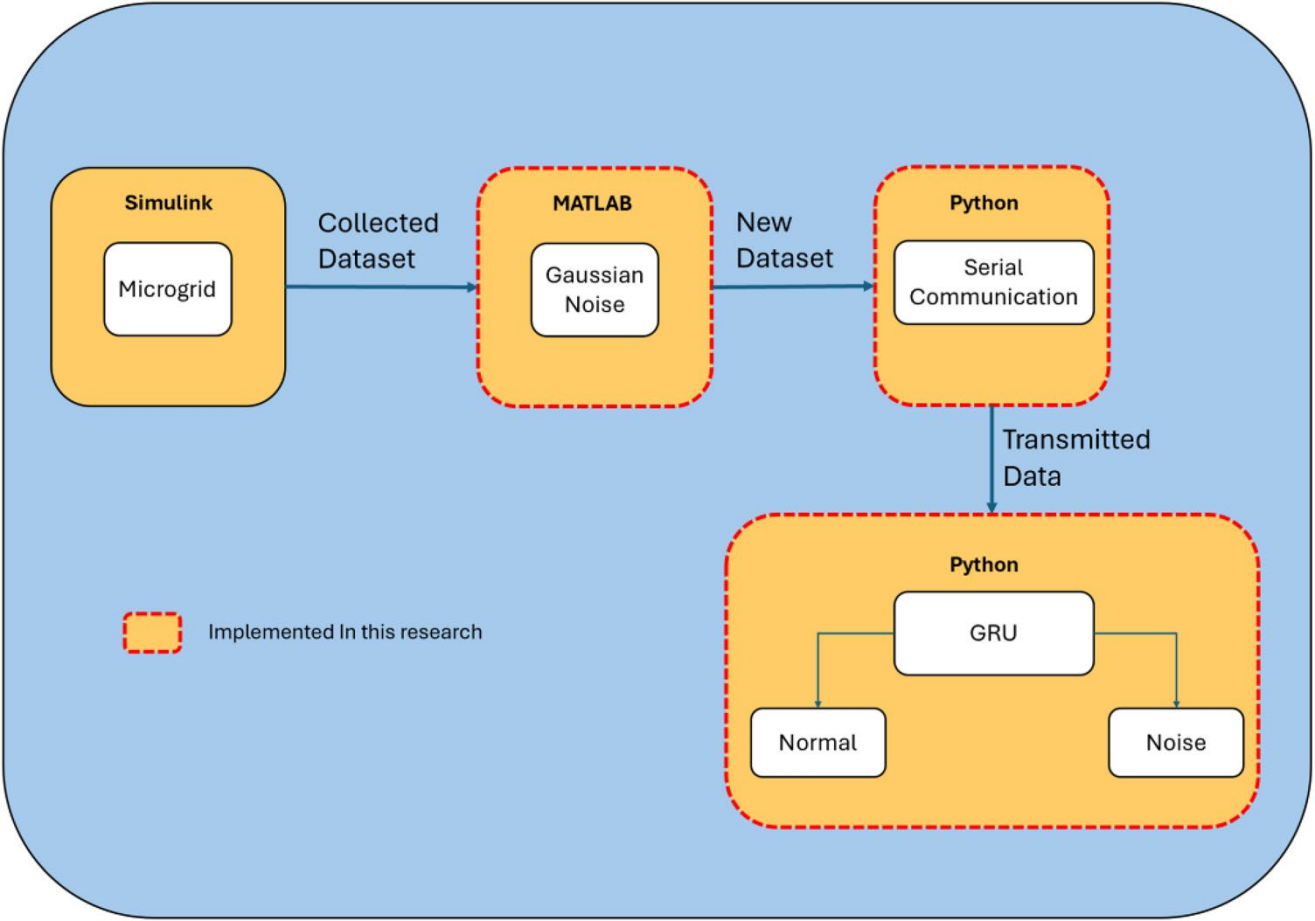
Noise classification simulation model.

The proposed architecture Noise Classification Simulation Model (NCSM) utilized in this research is depicted in Fig. 2. Initially, the simulation model named Simplified Model of a Small-Scale Micro-Grid^64^ is executed to generate a microgrid dataset that is stored in MATLAB matrix form. The generated dataset comprises different parameters measured by six sensors, which are solar power, secondary power, power load, battery power, battery state of charge, and battery ampere-hour over a 24-h simulation period. Gaussian white noise is added to the dataset to generate a new data dataset that includes noisy data and data without noise. The new data is transmitted using a serial communication model to simulate a real-time transmission of sensor data. The GRU model performs anomaly detection and classification of the emergent dataset. Both serial communication model and GRU are implemented in Python.

### Data generation model

**Figure 3:**
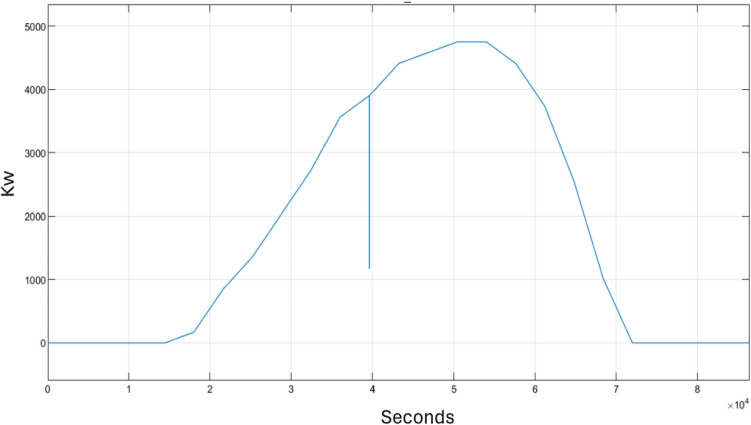
Solar power.

The dataset was generated from a microgrid simulation-based model reported in Ref.^64^. It consists of renewable energy sources components such as solar power, a power grid, a storage battery, a controller, and three houses. The purpose of the microgrid simulation model is to simulate power generation using solar panels, the storage of power using battery storage, and power consumption by the three households. It also includes grid power, used as secondary power generation when the battery storage runs out power, and involves a controller to switch between batteries storage and secondary power. The main objective of this simulation is to simulate the dynamics of balancing power generation and consumption over a 24-h period. There are six sensors to measure the dynamics of the microgrid model, which are solar power, secondary power, power load, battery power, state of charge, and battery ampere-hour. Figure 3 depicts the sensor readings of solar power generated over 24 h. The y-axis denotes the power generated in kilowatts (kW), while the x-axis corresponds to time measured in seconds starting at the second one stops at 86,400 s. It is observed that the solar panels generate power only between approximately 5 a.m. (18,000 s) and 7 p.m. (68,400 s) due to the presence of sunlight. The generated power peaks at 5 kW from 2 p.m. (50,400 s) to 3 p.m. (54,000 s).

**Figure 4:**
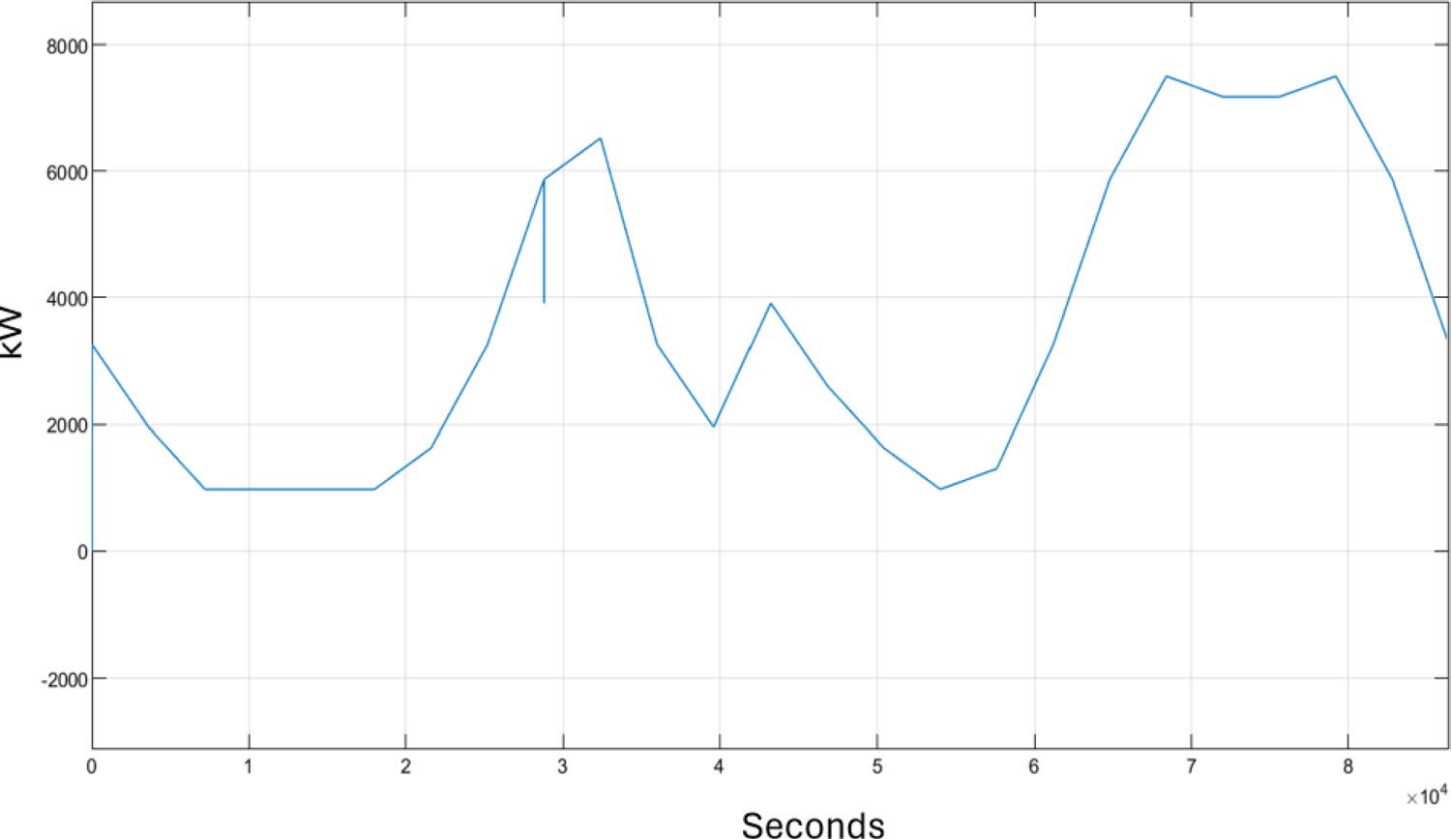
Load of three household.

Figure 4 shows the performance of the electric power load of the three households over 24 h, measured in kW. The load exhibits fluctuations throughout the simulation period due to the change in electricity demand. An increase in the load signifies a rise in power consumption, whereas a decrease indicates a reduction in consumption. The consumption pattern reaches three peaks at 9 a.m., 7 p.m., and 10 p.m., corresponding to periods when the electricity demand experienced fast increase. These peaks are attributed to increased activities typically occurring during these specific times.

**Figure 5:**
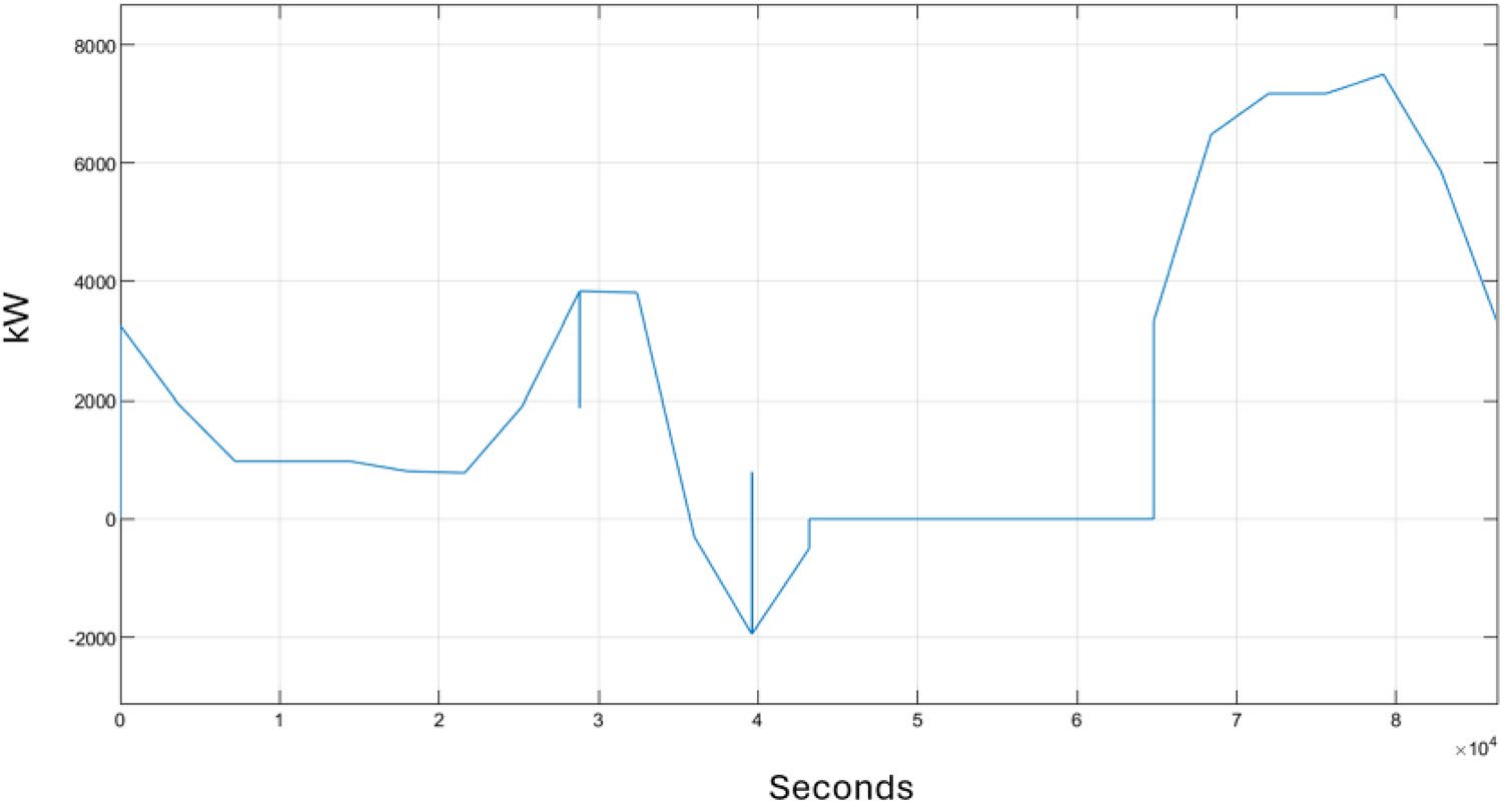
Power of the battery.

Figure 5 illustrates the performance of the storage battery in kW. The battery actively discharges from 1 a.m. to 12 p.m. and from 6 p.m. to 12 a.m., with fluctuations in power output corresponding to variations in demand and the battery’s charging cycles. A zero-power reading indicates that the battery has been deactivated by its controller, during which time secondary storage systems supply electricity to the households. Concurrently, the battery undergoes recharging, explaining why its power output reaches a peak once it resumes operation.

**Figure 6:**
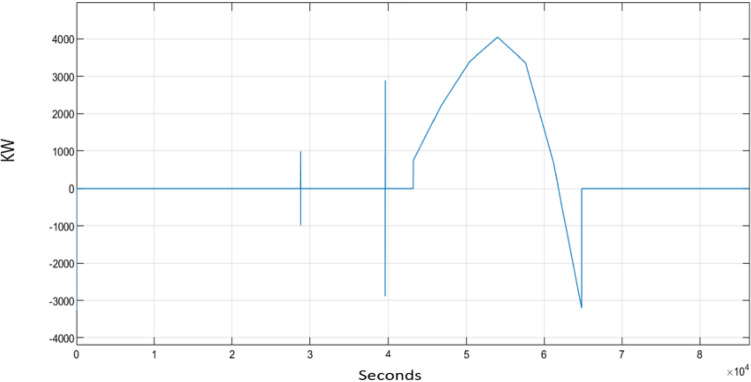
Secondary power.

Figure 6 shows the performance of the secondary power source in kW, as recorded by a sensor. From 12 p.m. to just before 6 p.m., the battery is deactivated, therefore secondary power is needed at that period of time. That is why secondary power output reaches its peak due to the microgrid’s reliance solely on the secondary power source (grid power). However, outside that time interval, the second power records no output since the battery is activated.

**Figure 7:**
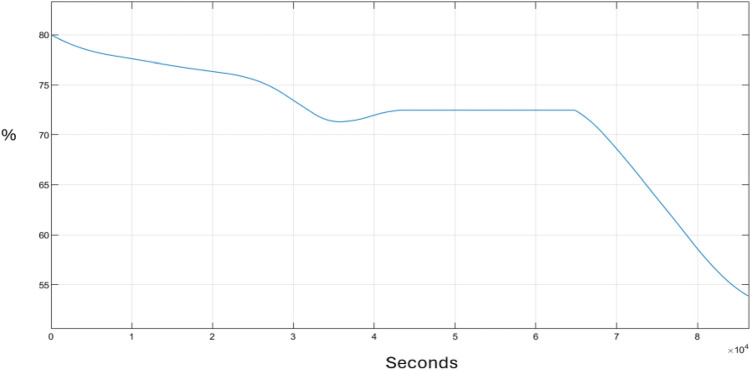
Battery SOC.

Figure 7 depicts the performance of the State of Charge (SOC) of a storage battery over 24 h. The SOC indicates the proportion of electrical energy currently stored in the battery, expressed as a percentage. The y-axis denotes the percentage of the battery’s charge, while the x-axis represents time measured in seconds. The battery’s charge was maintained between 80% and just above 50% throughout the simulation. Moreover, the battery discharged gradually; however, between 12 p.m. and 6 p.m., as illustrated by the plateau in the graph, the SOC remained constant because, at this time, the battery was deactivated by the controller, and the system relied on the secondary power source. After 6 p.m., there is a noticeable drop in the SOC due to a high demand for electricity in the system.

**Figure 8:**
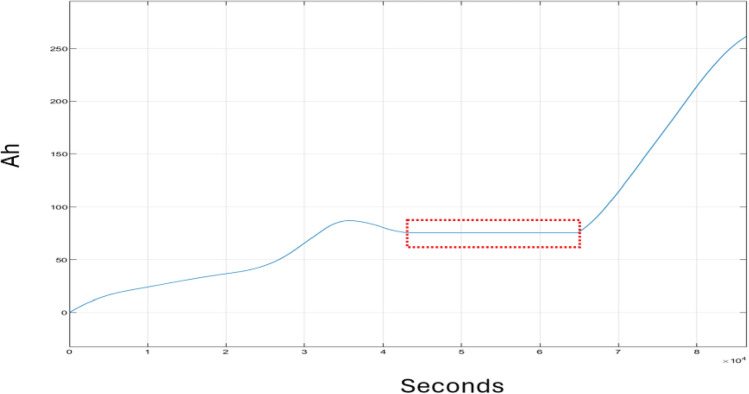
Battery Ah.

Figure 8 exhibits the performance of the SOC of the battery, quantified in ampere-hours (Ah), throughout the simulation period. The graph’s trajectory initiates with a marginal ascent in Ah, suggesting the battery is in charging mode. From 12 p.m. to 6 p.m., the graph’s progression stabilizes, indicative of the battery controller suspending the charging process. Subsequently, just after 6 p.m., there is a pronounced escalation in Ah, signifying that the controller has reinstated the battery’s charging mode.

The dataset generated from the microgrid simulation model consists of six features; each feature represents a sensor measurement. Table 2 provides a brief overview of sensor readings (features). Solar power, secondary power, power load, and battery power are measured in kW, while battery SOC is represented as a percentage, and battery Ah is measured in Ah.

**Table 2:** Dataset features.

Feature name	Unit
Solar power	kW
Secondary power	kW
Power load	kW
Battery power	kW
Battery SOC	%
Battery Ah	Ah

### Noise implementation

The noise is implemented based on additive white Gaussian noise (AWGN). It can be mathematically modeled using Eq. (1)^65^. Where P($n$) is the probability density function for the noise ($n$), $\pi$ *Denotes* the Pi, ${\sigma }^{2}$ is the variance of the noise, $e$ is the base of the natural logarithm is approximately equal to 2.71828. The equation is given as:

1 $$p(n)=\frac{1}{\sqrt{2\pi {\sigma }^{2}}}{e}^{-\frac{{n}^{2}}{2{\sigma }^{2}}}$$

The Signal-to-Noise Ratio (SNR) utilizes the ratio of the power of the signal (${P}_{signal}$), and the power of noise (${P}_{noise}$) as shown in Eq. (2):

2 $${SNR}_{linear}=\frac{{P}_{signal}}{{P}_{noise}}$$

The noise’s power is directly related to the variance. ${\sigma }^{2}$ is presented in Eq. (3):

3 $${P}_{noise}={\sigma }^{2}$$

Equation (4) is used to convert the SNR to decibels (dB):

4 $${SNR}_{dB}=10 {log}_{10}({SNR}_{linear})$$

The SNR values used in this study are − 10, − 30, − 50 and − 70. All are negative SNR values because a negative SNR indicates that the power of noise is predominant. Consequently, each sensor reading was influenced by a similar SNR value. As a result, the new dataset features five labels, as detailed in Table 3.

**Table 3:** Dataset label.

SNR value	Label number
− 10	1
− 30	2
− 50	3
− 70	4
No noise	0

### Serial communication model

This section discusses the design of the serial communication model employed in this study. The purpose of implementing such a model is to simulate a real-world scenario of a microgrid system in which sensor data is transmitted through the communication channel in real-time. The serial communication model is implemented using the serial Python library. Algorithm 1 outlines the implemented serial communication model. Initially, the input phase of the algorithm includes essential variables: the filename, a string variable utilized to specify the path of the CSV file containing the dataset. *PORT* is another string variable utilized to assign the port number for communication. *BAUD_RATE* is utilized to determine the maximum rate for transmitting bits per second. *SEND_TIMEOUT* establishes a timeout for sending data, and *RECV_TIMEOUT* delineates the waiting time in seconds for receiving data. The anticipated output is the specified data transmitted to the serial port, in this instance, the six features previously discussed. The primary objective of the algorithm is presented during the procedure phase. It establishes a communication channel through an assigned serial port and uses the variables from the input phase, namely *PORT*, *BAUD_RATE*, and *SEND_TIMEOUT*. Once the connection is established, the dataset’s CSV file is opened, and each record within the data is read. Subsequently, each record is transformed into a byte-encoded string within a loop because serial communication deals with byte streams. Following this transformation, the encoded string is transmitted through the serial port. Ultimately, the file object is closed, and the port remains open, awaiting coming data for a predetermined duration before it closes.

**Figure:**
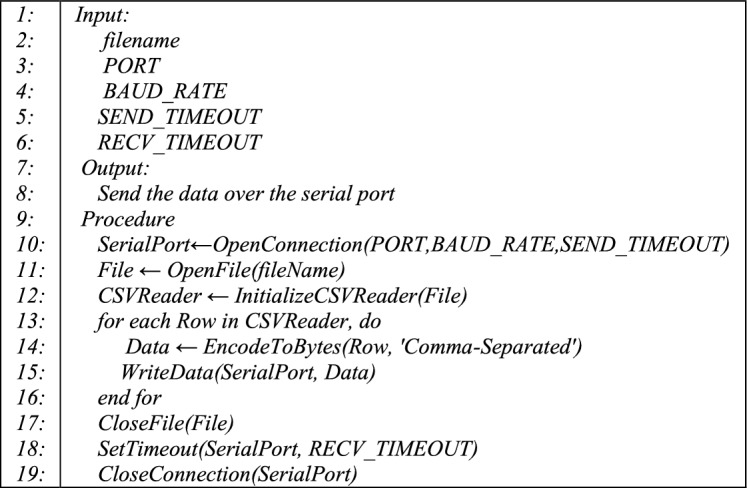
Algorithm 1. Serial communication.

### GRU model

The GRU is an advanced recurrent neural network form. It was first proposed in 2014 by Cho et al. as a simple form of long short-term memory^66^. GRU is a gating method used to manage sequential data and regulate information flow through a gating structure. Both GRU and LSTM are used to discover long-term dependencies in sequential data. GRU is similar to LSTM with fewer gates, which are the update gate (combines the input gate and forget gate) and the reset gate, whereas LSTM has three gates: the input gate, forget gate, and output gate. In GRU, there is one hidden memory called hidden state memory; LSTM contains two memories: short-term and long-term. GRU uses its gates to manage the information flow through the network and decide which information to remember or forget at each stage. The update gate determines how much previous information should pass to the next state. The reset gate is used to determine the critical data from the previous state and decides how much of the previous information to forget. Therefore, a GRU model can remember valuable information while avoiding the risk of forgetting significant details^67^. GRU models can be used in different domains that deal with sequence classification tasks, for instance, sentiment analysis, spam detection, and subject categorization^68^. The GRU model contains fewer gates and parameters compared with the LSTM model, therefore it is generally easier and faster to train^69^. The GRU model used in this study consists of one input layer, two hidden layers, and one output layer. The GRU model is considered a type of RNN, and the size of the input layer depends on two factors the sequence length and number of features. In this case, the sequence length equals 10 and the number of features is 5. The sequence length refers to the number of previous observations (data points) that the model is used in order to predict at any given time. The hidden layer consists of two layers, and each layer has 50 nodes, which are used to process the input data to capture their patterns and behavior so the model can learn the relationship between data to use for prediction. The output layer consists of a dense layer with nodes the same size as the number of unique classes. The SoftMax activation function converts the raw prediction data, usually logits, into probabilities. Logits are real, unnormalized outputs of the last layer of the network. The Softmax function is used to normalize logits by taking the exponential of each logit and dividing it by the sum of the exponential of all logits. This process ensures that the output probabilities are distributed from 0 to 1 and that the sum of all probabilities is equal to 1. Then, the model selects the class of the highest probability as the predicted class. The equation of SoftMax function is shown in Eq. 5 where $e$ is a constant number that is approximately equal to 2.71828 and ${z}_{i}$ is the logit, and $N$ is the total number of classes.

5 $$Softmax\left({z}_{i}\right)=\frac{{e}^{zi}}{\sum_{j=1}^{N}{e}^{zj}}$$

**Figure 9:**
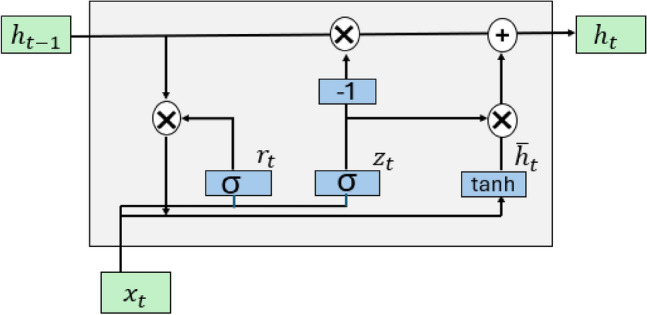
Gated recurrent unit block architecture.

Figure 9 illustrates the architecture of the GRU model. ${x}_{t}$ refers to the input $x$ at the current time $t$ and ${h}_{t-1}$ is the output of the previous hidden state at time $t-1$^70^. The reset gate is modeled mathematically as shown in Eq. (6):

6 $${r}_{t}=\upsigma \left({W}_{r}\cdot \left[{h}_{t-1},{x}_{t}\right]+{b}_{r}\right)$$

where ${r}_{t}$ represents the reset gate’s variable at time t. Rest gate is used to determine the amount of the previous hidden state that needs to be forgotten. $\upsigma$ is the sigmoid function, which normalizes the input to be ranged between 0 and 1. ${W}_{r}$ represents the weight associated with the rest gate, and ${b}_{r}$ refers to the bias term of the reset gate.

The update gate is presented in Eq. (7) as follows:

7 $${z}_{t}=\upsigma \left({W}_{z}\cdot \left[{h}_{t-1},{x}_{t}\right]+{b}_{z}\right)$$

where ${z}_{t}$ refers to the update gate’s variable at time t, $\upsigma$ is the sigmoid function, ${h}_{t-1}$ is the output of the previous hidden state at time t-1, and ${x}_{t}$ is the input of the current time, ${W}_{z}$ is the weight associated with the update gate, and ${b}_{z}$ refers to bias term of the update gate.

The candidate hidden state is calculated in Eq. (8):

8 $${\overline{h} }_{t}=\text{tanh}\left({W}_{h}\cdot \left[{{r}_{t}*h}_{t-1},{x}_{t}\right]+{b}_{h}\right)$$

The above equation calculates a candidate for the new hidden state where ${W}_{h}$ represents the weight. When ${r}_{t}$ is 0, the result of ${{r}_{t}*h}_{t-1}$ is 0, which indicates that the previous state will be ignored, and the model will focus on the new state. ${b}_{h}$ refers to the bias term of the candidate hidden state. Finally, Eq. (9) calculates the final activation.

9 $${h}_{t}=\left(1-{z}_{t}\right)*{h}_{t-1}+{z}_{t}* {\overline{h} }_{t}$$

A description of variables used in the methodology section is listed in Table 4.

**Table 4:** Summary of used variables.

Variable	Description
*P(*$n$*)*	The probability density function for the noise ($n$)
$\pi$	Pi which is approximately equal to 3.14159
${\sigma }^{2}$	The variance of the noise
$e$	Euler’s number
${SNR}_{linear}$	The Signal-to-Noise Ratio in linear scale
${P}_{signal}$	The power of the signal
${P}_{noise}$	The power of noise
${SNR}_{dB}$	The SNR is converted to decibels dB
$log$	Logarithm
CSV	Comma-separated values file
*filename*	String variable used to specify the path of the CSV file
*PORT*	String variable used to assign the port number for communication
*BAUD_RATE*	The maximum rate for transmitting bits per second
*END_TIMEOUT*	The timeout for sending data
*RECV_TIMEOUT*	The waiting time in seconds for receiving data
${z}_{i}$	The logit for class $i$
$N$	The total number of classes
${r}_{t}$	The reset gate at time $t$
$\upsigma$	The sigmoid function
${W}_{r}$	The weight associated with the rest gate
${h}_{t-1}$	The output of the previous hidden state at time $t-1$
${x}_{t}$	The input $x$ at the current time $t$
${b}_{r}$	The bias term of rest gate
${z}_{t}$	The update gate at time t
${W}_{z}$	The weight associated with the update gate
${b}_{z}$	The bias term of the update gate
${\overline{h} }_{t}$	The candidate hidden state at time t
${W}_{h}$	The weight associated to the candidate hidden state
${b}_{h}$	The bias term of the candidate hidden state
${h}_{t}$	The final activation at time t

## Experiment and result

This section discusses the experimental outcomes of this study. Initially, it delineates the experimental setup, including information on the hardware and software employed to achieve the claimed result. Subsequently, it presents the results derived from the proposed methodology’s training, testing, and evaluation.

### Experimental configuration

The experimental configuration encompasses the following components: a Simulink model, which incorporates the microgrid system; MATLAB, which is primarily utilized for gathering data and applying noise; and Python, employed for establishing serial communication and performing the GRU training and testing. Table 5 lists the environmental configurations utilized for conducting the experiments. Simulink is a platform that models and simulates multidomain dynamical systems^71^. It operates as a graphical programming language, distinct from a scripting language, making it suitable for modeling and simulation, particularly with complex systems. The reason for using Simulink in this study is that the “Simplified Model of a Small-Scale Micro-Grid” is modeled with it. MATLAB is a high-level programming language widely used by scientists and engineers^72^. MATLAB is applied to store data generated by the microgrid model in matrix format. Furthermore, it is utilized to add white Gaussian noise to the generated dataset. Python, another high-level programming language, implements the serial communication model, trains and evaluates the GRU model, and executes the experiments. The comprehensive experiments were executed on a personal computer that equipped with NVIDIA® GeForce RTX™ 4090 GPU with 24 GB of memory. The processor is an Intel Core i9 14900 K with speeds up to 5.8 GHz. The computer has 32 GB of RAM and 1 TB of hard-disk storage.

**Table 5:** Environmental setup.

Tool name	Type	Purpose of utilization
Simulink	Software	Employed for data acquisition from the microgrid model
MATLAB	Software	They store the data from the Simulink platform and implement the Gaussian noise algorithm on the collected data
Python	Software	She was deployed to train and assess the GRU model and perform the experiment in real-time
Personal computer	Hardware	A sophisticated personal computer with high-performance components utilized for experimental execution, detailed as follows GPU: NVIDIA® GeForce RTX™ 4090 with 24 GB Processor: Intel Core i9 14900 K with speeds up to 5.8 GHz RAM: 32 GB Storage: 1 TB SSD

### GRU training and evaluation

The GRU model was trained with the collected dataset, as discussed in “Problem statement”. The data preprocessing stage mainly focuses on preparing the GRU model for the training and testing phases. The GRU model was trained using two approaches. Initially, the model undergoes training based on a data partition approach in which the entire data set is divided into two subsets in an 80/20 ratio. Thus, 80% is utilized for training, and the remaining 20% is designated for testing. Furthermore, GRU is also trained using a fivefold cross-validation technique. This approach randomly splits the dataset into five equal or approximately equal parts. Four subsets are employed for training, while the fifth subset is reserved for testing.

#### Partition approach

**Figure 10:**
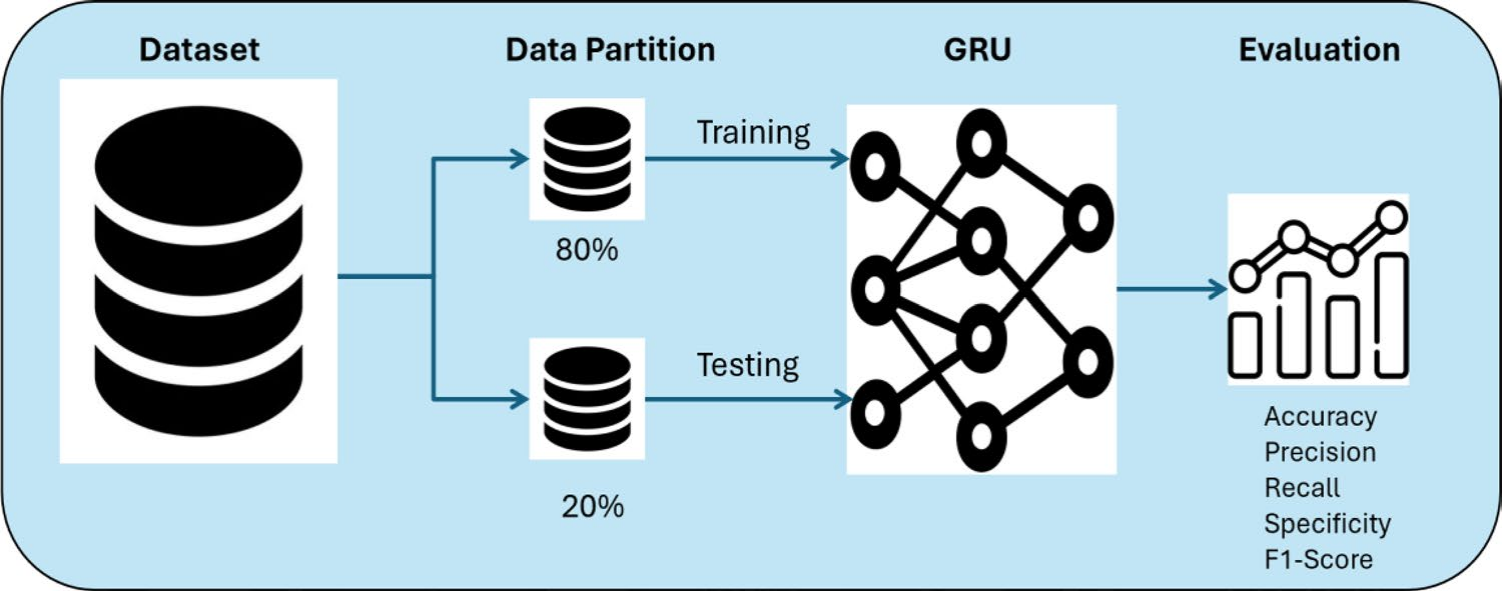
Partition approach.

As mentioned, the data were divided into two parts for the partition approach: 80% for training and 20% for testing, as shown in Fig. 10. The training set is used to train the GRU model. The testing set is kept for evaluating the model. The GRU is evaluated based on well-known evaluation metrics, which are precision, recall/sensitivity, specificity, F1-score and classification accuracy. TP is the true positive sample, TN is the true negative sample, FP is the false positive sample, and FN is the false negative sample^73,74^. The evaluation metrics are shown in Eqs. (10–14).

10 $$\text{Precision }= \frac{TP}{TP+FP} \times 100$$

11 $$\text{Recall}/\text{sensitivity }= \frac{TP}{TP+FN} \times 100$$

12 $$\text{Specificity }= \frac{TN}{TN+FP} \times 100$$

13 $$F-1\text{ score }= \frac{TP}{TP+\frac{FP+FN}{2}} \times 100$$

14 $$\text{Classifier Accuracy }= \frac{TP+TN}{TP+FP+TN+FN} \times 100$$

**Figure 11:**
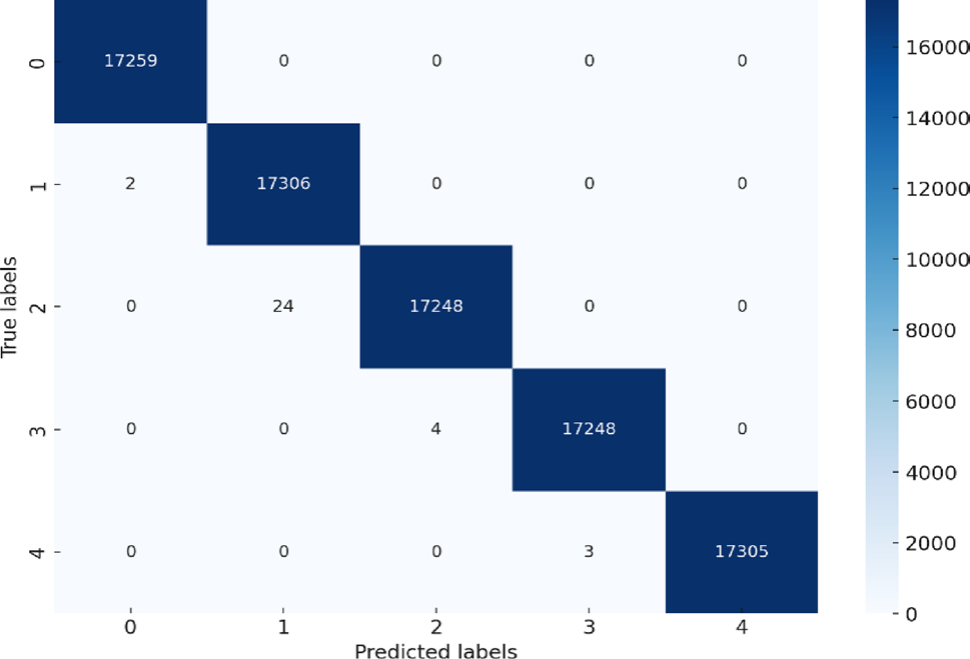
Confusion matrix for testing.

The confusion matrix for testing is shown in Fig. 11, which is used to calculate the accuracy of the classification model. This is done by comparing the predicted values against the actual values. As shown in the figure, there are five classes, class 0, class 1, class 2, class 3, and class 4, with the following labels respectively: 0, − 10, − 30, 50, and − 70, and each class represents a signal with an SNR value, except for class 0, which refers to a normal signal. The x-axis is used for predicted classes, and the y-axis is used for the true class. The diagonal of the matrix (shown in dark blue) refers to the number of correct predictions. It can be observed that 17,259 instances of the data were predicted correctly as class 0, and there was no misclassified prediction. 17,306 instances were classified correctly as class 1, but two instances were misclassified as class 0. 17,248 instances were predicted correctly as class 2; however, 24 were mispredicted as class 1. 17,248 instances were predicted correctly as class 3, but 4 were predicted incorrectly as class 2. Finally, 17,305 instances were classified correctly as class 4, but 3 were predicted incorrectly as class 3.

The comprehensive performance analysis of the GRU model for each class, utilizing testing data, is listed in Table 6. The accuracy metrics presented in the table consist of the following: precision, recall (also known as sensitivity), specificity, and f1-score, each providing insightful analysis of the model’s classification efficacy. Precision calculates the ratio of true positive classifications to the combined total of true positive and false positive classifications. The model achieves a noteworthy precision score, exceeding 99.86%. Recall (sensitivity) is measured by dividing the true positive predictions by the sum of the true positive and the false negative. It reveals that the model produced an exceptional recall score. Specificity evaluates the model’s accuracy in correctly identifying true negatives, yielding a specificity score exceeding 99.97%. The F1-score is calculated by ascertaining the harmonic mean of precision and recall. With an F1-score performance surpassing 99.90%, there is a demonstrated equilibrium between precision and recall. Accuracy determines the proportion of the correct predictions of the model compared with total predictions. The model demonstrates high accuracy in classifying classes, as indicated by Table 7. The classification accuracy of 99.96% indicates that the model achieved the desired result. Additionally, the training loss and the average validation loss scores are very low, demonstrating that the model underwent a successful training process.

**Table 6:** Evolution of GRU model using testing data.

Class #	Label	Precision	Recall (sensitivity)	Specificity	F1-Score
0	0	99.99%	100%	100%	99.96%
1	− 10	99.86%	99.99%	99.97%	99.92%
2	− 30	99.98%	99.86%	99.99%	99.92%
3	− 50	99.98%	99.98%	100%	99.98%
4	− 70	100.00%	99.98%	100%	99.99%

**Table 7:** GRU accuracy.

Metric	Value
Classification accuracy	99.96%
Training loss	0.0021
Validation loss	0.0018

#### k-fold cross-validation approach

**Figure 12:**
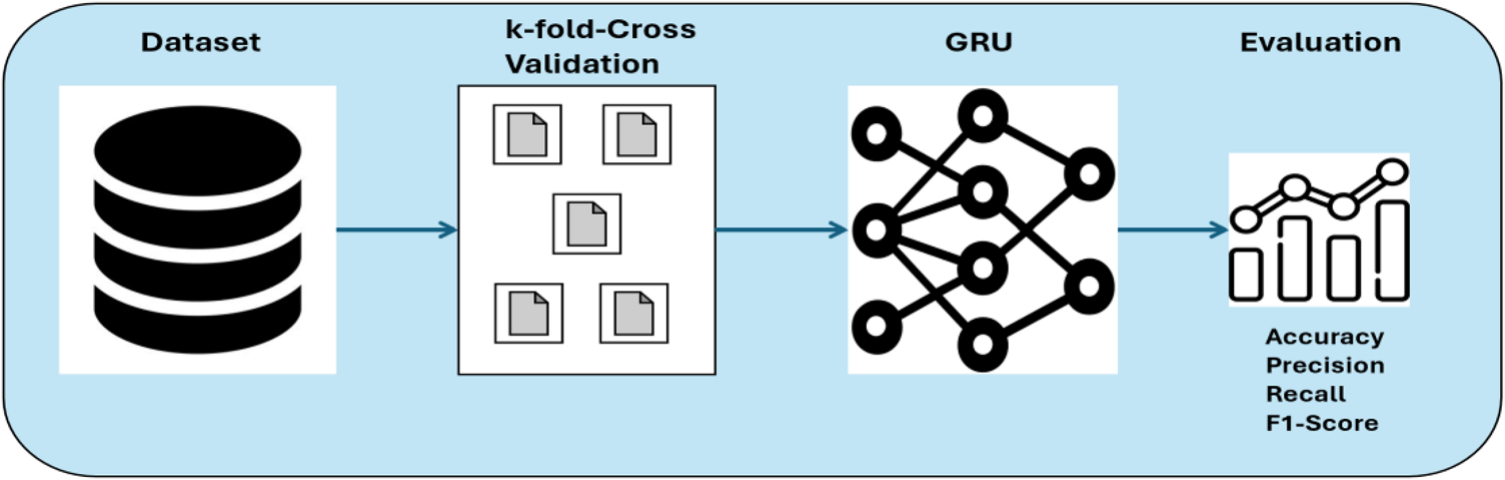
k-Fold cross-validation approach.

Besides the partition approach, the GRU model is assessed using a fivefold cross-validation method. This technique helps to evaluate the model’s generality, which results in mitigating overfitting. Figure 12 illustrates the procedure of employing k-fold cross-validation. The entire dataset is divided into five subsets randomly, which are also called folds. The GRU model iterates five times. In each iteration, four sets are used for training and the fifth is used for testing. Then, the accuracy metrics are calculated to evaluate the model.

Correspondingly, Table 8 presents the evaluation metrics for the GRU model to assess the performance of this classification prediction of the SNR value. Notably, the training loss is calculated for each fold, indicating that the loss value is minimal and close to 0. The overall accuracy using fivefold cross-validation is 99.95%. Regarding the accuracy metrics, namely, precision, recall, and F1-score, the results enumerated in the table confirm the generality of the GRU model without experiencing any incidence of overfitting.

**Table 8:** Evolution using fivefold-cross validation.

Fold number	Loss	Precision	Recall	F1-Score
1	0.0020	99.95%	99.95%	99.95%
2	0.0015	99.97%	99.97%	99.97%
3	0.0021	99.94%	99.94%	99.94%
4	0.0017	99.97%	99.97%	99.97%
5	0.0018	99.95%	99.95%	99.95%

In addition, the regularization methods are employed to ensure the GRU model’s generalization capability and mitigate overfitting. The report of using the Ridge regularization method and the dropout technique is included in this study. Ridge regularization is applied to balance the GRU parameter weights across each feature to ensure the generality of the model and to prevent overfitting^75^. It motivates the model to use a maximum number of features rather than relying on a minimum feature. Furthermore, the dropout method ensures that the GRU uses a large number of neurons, rather than focusing on limited neurons. This is done by randomly deactivating several neurons during training, which forces other neurons to learn. Consequently, it maintains the generality of the GRU model and eliminates overfitting. The classification accuracy after using these regularization methods is 99.91%.

### Comparing with different models

**Figure 13:**
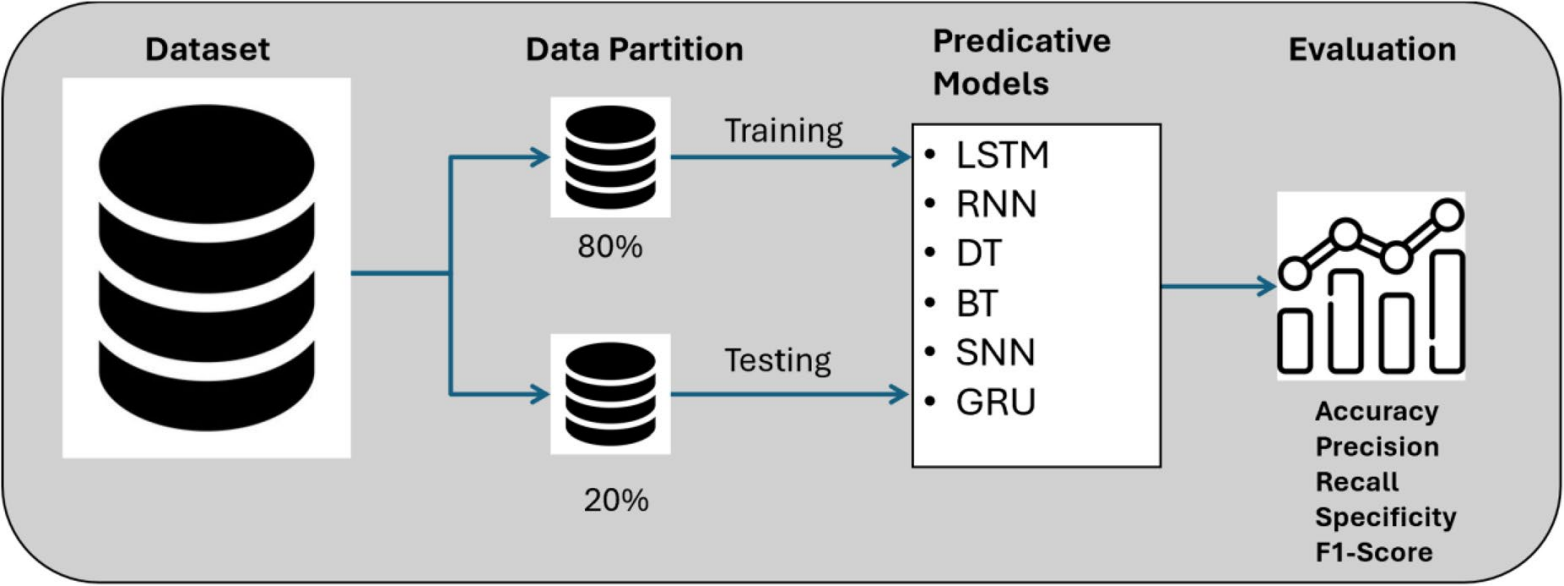
Training and testing of different ML models.

To ensure the credibility of the evaluation results obtained in this research, the findings were compared with prevalent predictive models, such as LSTM, conventional RNN, decision tree (DT), bagged tree (BT), and shallow neural networks (SNN). The dataset was split into an 80/20 ratio, similar to the partitioning procedure shown in Fig. 13. The overall accuracy of each model is shown in Table 9. The reason for contrasting these results with the LSTM and RNN models is that their designs are similar to GRU, and both belong to the recurrent neural network family. LSTM yields a comparable result to those of the GRU, thereby confirming the validity of the GRU outcomes. The comparison with the decision tree and the Bagged Tree models is motivated by their popularity in classification. Finally, the GRU was evaluated against shallow neural networks to determine the impact of conventional neural network models on noise classification, where it generated the lowest scores in the table.

**Table 9:** Accuracy of various predictive models.

Class label	Accuracy
LSTM	99.52%
RNN	98.70%
Decision tree	96.93%
Bagged tree	99.90%
Shallow neural networks	95.63%
GRU	99.96%

**Figure 14:**
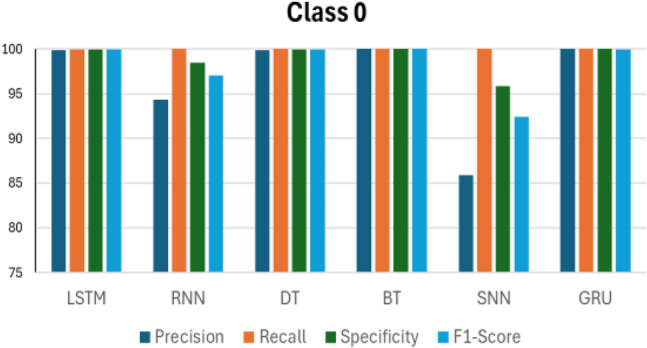
Performance in Class 0.

**Figure 15:**
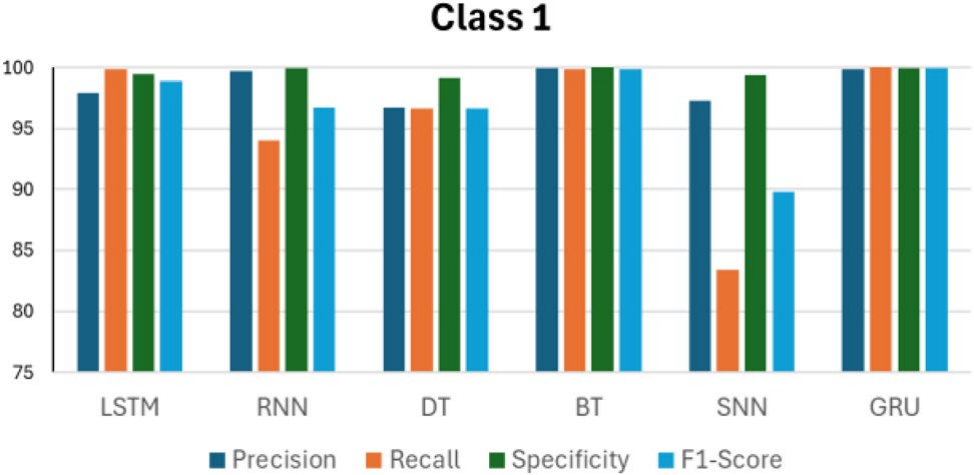
Performance in Class 1.

**Figure 16:**
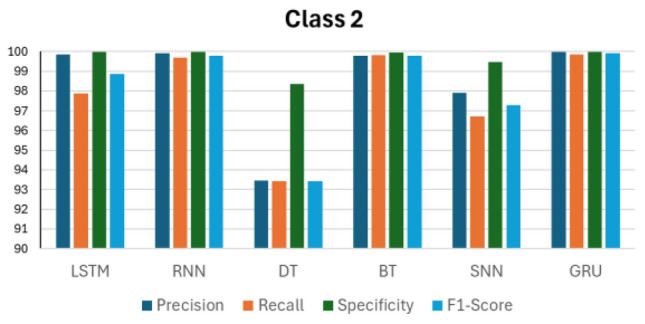
Performance in Class 2.

**Figure 17:**
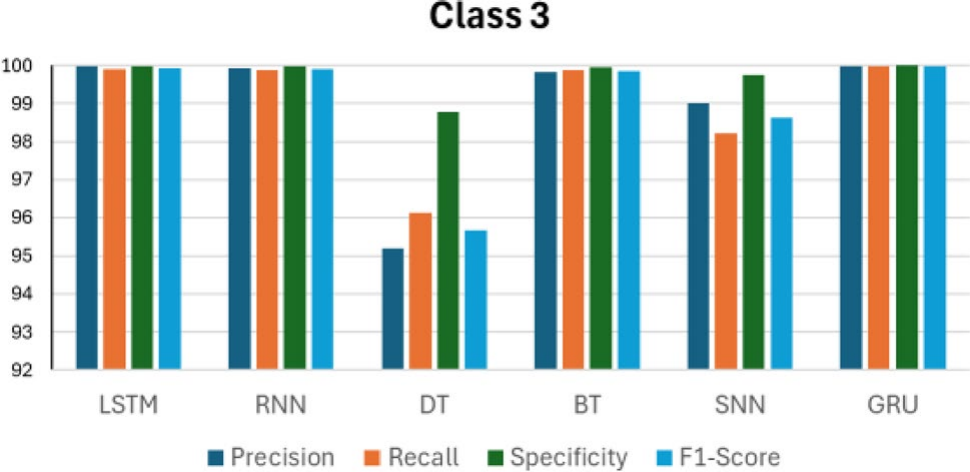
Performance in Class 3.

**Figure 18:**
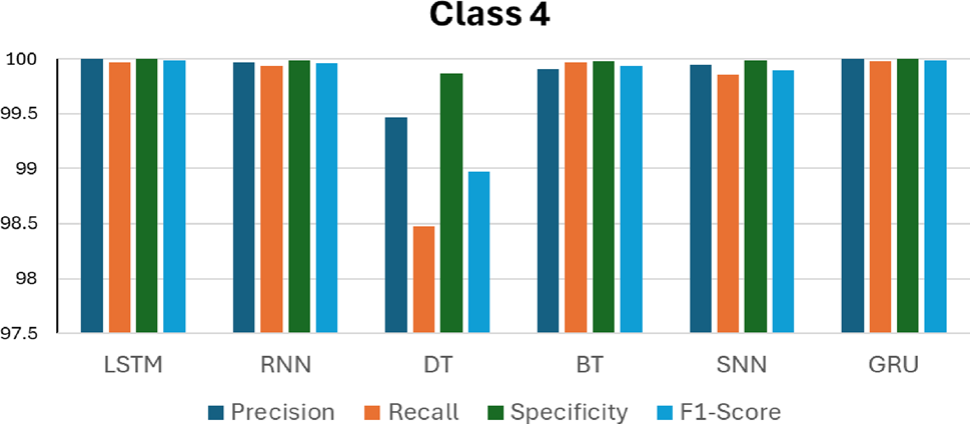
Performance in Class 4.

Figures 14, 15, 16, 17 and 18 compare the accuracy metrics: precision, recall, specificity, and f1-score of each model for classes 0–4. The objective is to analyze the performance of each model relative to the GRU. The LSTM model performs well with each metric in predicting classes 0, 3, and 4. RNN shows perfect results in predicting classes 2, 3 and 4. The decision tree achieves perfect accuracy in predicting class 1. The bagged tree performs well in predicting any class. Shallow neural networks yield better results in predicting class 4. Figure 14 shows the performance of LSTM, RNN, DT, BT, SNN, and GRU models in predicting class 0, measured by evaluation metrics. LSTM, DT, BT, and GRU show comparable performance with each metric, however RNN records a precision value of less than 95%, and SNN presents precision of less than 90%. RNN and SNN record high recall values. Figure 15 illustrates the performance of ML models when predicting class 1. This time, GRU and BT show slightly better performance compared with LSTM and DT. RNN shows high precision and specificity values but slightly lower recall and F1-score. SNN presents a recall value less than 85% and its F1-score value is under 90%. RNN, BT, and GRU respond similarly in predicting class 2. However, it is clear that DT indicates the lowest performance compared with all models in predicting class 2. The same applies to predicting class 3, but this time LSTM performance enhances slightly.

According to Figs. 14, 15, 16, 17 and 18 and the previous discussions, the GRU model outperforms LSTM, RNN, DT, BT, and SNN. It maintains high results in all evaluation metrics when predicting each class. The GRU does not experience any noticeable drops in evaluation metrics in predicting any class. Furthermore, the GRU model indicates a stable response in predicting all classes, as confirmed by its robust evaluation metrics. The superior performance of the GRU model is due to its effectiveness in working with sequential data, which is the nature of the collected data from the simulation model, as done in this research. Additionally, the GRU has a simpler architecture than LSTM, which enhances its efficiency.

## Conclusion

In conclusion, a microgrid system is used to manage energy efficiency by relying on renewable energy sources. The noise interference presents a challenge to networked systems such as networked microgrids, which can lead to distorted networked traffic data transmitted over the network. To address noise challenge, we proposed a Noise Classification Simulation Model (NCSM) that use the power of deep learning to predict noise in data in real-time. The proposed model was achieved by several steps. In the first step, Gaussian white noise was applied to the microgrid dataset, which helped us build an emergent dataset that has noisy data and data without noise. In the second step, the serial communication model was implemented to transmit sensor data in real-time to simulate the real scenarios. In the third step, the GRU model was implemented for predicting the SNR values. The performance of GRU model was evaluated based on two approaches, which are testing data and fivefold cross-validation to ensure the reliability of the model. GRU scored 99.96% classification accuracy. Finally, the classification performance of GRU model was compared with well-known machine learning models to confirm its validity, which are LSTM, RNN, DT, BT, and SNN. The experimental results confirmed that GRU achieved superior performance compared with others. Therefore, the NCSM is promising in predicting the SNR values captured from networked traffic data. By minimizing the impact of noise, the NCSM helps the development of networked microgrids and might be useful in showing their potential for enhancing the sustainability and resilience of the microgrid. However, this study concentrated on noise that is generated based on Gaussian white noise. Therefore, in future work, we suggest studying various communication channel disturbances, such as Fading channel, Multipath Fading channel, or Rician channel. Another future work is to test our model for detecting cyberattacks that can be established through the communication network in real-time. In addition, the model should distinguish between noise produced by microgrid hardware and cyberattacks created by invaders.

## Data Availability

The datasets used and/or analysed during the current study are available from the corresponding author upon reasonable request.
